# Protonation- and substrate-regulated dimer opening couples brain-type creatine kinase to vesicular and actin-remodeling membranes

**DOI:** 10.64898/2026.08.04.742861

**Published:** 2026-08-09

**Authors:** Samantha Gies, Nolan K. McLaughlin, Ahmed Shubbar, Chen Kong, Tianqi Wu, Sana Khan, Alessandro Ustione, Ian Miller, David W. Piston, Mahmoud Moradi, Michael L. Gross, Reza Dastvan

## Abstract

Brain-type creatine kinase (CK-BB) buffers local ATP demand through reversible phosphotransfer between ATP and phosphocreatine, yet how this soluble metabolic enzyme couples to dynamic membrane compartments remains unclear. Here, we integrate immunofluorescence microscopy, DEER spectroscopy, hydrogen-deuterium exchange and native mass spectrometry, DEER- and AlphaFold-guided modeling, and long-timescale molecular dynamics to define the acid- and substrate-regulated conformational landscape of human CK-BB. Acidification redistributes endogenous and recombinant CK-BB from a diffuse cytosolic pool to punctate vesicular structures and membrane ruffles. DEER and modeling reveal a spatially asymmetric dimer in which the convex surface remains comparatively restrained, whereas the concave catalytic-regulatory surface samples pH- and substrate-dependent conformational intermediates. We identify progressive dimer opening as a novel regulatory mechanism by which acidic pH and substrate binding increase dynamics across the convex surface and NTD–NTD interface and sensitize the His191/Ser199 module to graded pH-dependent exchange. Substrate binding buffers acid-induced deprotection while preserving localized dynamics near this regulatory interface. Together, these data support a model in which protonation and substrate occupancy generate membrane-competent CK-BB conformations, coupling local ATP regeneration to curved vesicular and actin-remodeling membranes.

## Full Text Availability

The license terms selected by the author(s) for this preprint version do not permit archiving in PMC. The full text is available from the preprint server.

